# Supplementary material for: Human tactile sensing and sensorimotor mechanism: from afferent tactile signals to efferent motor control
Source: Nat Commun. 2024 Aug 10;15:6857. doi: 10.1038/s41467-024-50616-2 (PMC11316806; doi:10.1038/s41467-024-50616-2)
Supplement: Supplementary file 1 — Supplementary Information [file 41467_2024_50616_MOESM1_ESM.pdf]

Supplementary material for 'Human tactile sensing and sensorimotor mechanism from afferent tactile signals to efferent motor control'

*Corresponding author. Email: lren@jlu.edu.cn and g.wei@salford.ac.uk

This PDF file includes:

Summary of the FE Human Hand Model

Detailed Process for Deriving the Sensorimotor Functions and Parameter Determination:

Fig. S1. The microneurography test and the experimental results.

Fig. S2. In-vivo grasping experimental results based on all six human subjects

Fig. S3. Experimental setup of the in-vivo grasping tests.

Fig. S4. The neural activation levels predicted based on summarized transduction function compared with those computed based on the electromyography signals captured from the human subject.

Fig. S5-8. The neuromorphic afferent tactile signals

Fig. S9. The neural activation level of the muscle synergy and contact pressure during the active and reactive grasping implemented by ATSS. The biological and neuromorphic 2nd order tactile afferent signals were also presented.

Fig. S10. The measured neural activation levels and those predicted based on summarized transduction function.

Fig. S11. The contact pressure on the index finger of human and biomimetic hand during active and reactive grasping.

Fig. S12. The hardware setting of the artificial tactile sensory system.

Fig. S13. The computing of discrimination accuracy base on the neural features of spiking rate and Victor-Purpura distance.

Fig. S14. The artificial tactile sensory system mounted on the Kuka robotic arm.

Table. S1-5. The values of poles of the summarized transduction functions representing the sensorimotor control algorithm during active grasping. The cylindrical, spherical grasping and precision gripping were performed for five times and the corresponding transduction function was summarized.

Table. S6-10. The values of poles of the summarized transduction functions representing the sensorimotor control algorithm during reactive grasping. The cylindrical, spherical grasping and precision gripping were performed for five times and the corresponding transduction function was summarized.

Table. S11-20. The transduction functions extracted from the neural activation levels of the other five human subjects.

Table. S21. The gender and age of all the human subjects.

Table. S22. Victor-Purpura Distances Between Baseline Cylinder/Spherical Object (Diameter: 100mm) and Others.

Supplementary Materials:

Summary of the FE Human Hand Model

In our research, we have utilized a sophisticated and subject-specific Finite Element (FE) model of the human hand [1]. This model is central to our study, enabling us to simulate the biomechanical aspects of active touch with high accuracy. Below is a summary of the model's structure, material properties, and the validation process:

Anatomical Structure: The FE model incorporates a detailed representation of the human hand anatomy, including bones (phalanges, carpal, and wrist bones), ligaments, tendons, subcutaneous tissue, and skin. These components were reconstructed from CT and MRI scans of a specific subject, providing a highly realistic and anatomically accurate model.

Material Properties: Skin: Modelled as a heterogeneous, anisotropic, and viscoelastic material, capturing the complex behaviour of skin under mechanical stress. Bones: Treated as isotropic linear elastic materials with specific Young's modulus and Poisson's ratio. Ligaments and Tendons: Simulated using spring elements to represent their supportive role in joint movement. Subcutaneous Tissues: Characterized with properties that allow for realistic simulation of soft tissue deformation.

Deformability and Biomechanics: The model is designed to be deformable, simulating the biomechanical behavior of the hand during interactions, such as grasping or touching. This includes the accurate representation of joint movements, tissue deformation, and response to external forces.

Validation Process: The model underwent rigorous validation against in-vivo experimental data. This included comparing predicted mechanical responses, such as contact pressure and area, with actual measurements from hand interactions.

Sensitivity Analysis: We conducted sensitivity analyses to understand the influence of various material properties and loading conditions on the model's predictions.

Implementation in Current Research: In our study, the FE hand model is used to simulate the mechanical responses of the hand during active touch scenarios. The model's outputs, such as strain and stress distributions, are crucial inputs for our multi-level numerical model that predicts afferent neural signals.

This FE model of the human hand, with its detailed anatomical structure and validated biomechanical properties, plays a pivotal role in our research. It allows us to bridge the gap between biomechanical interactions and neural processing, enhancing our understanding of tactile perception.

Detailed Process for Deriving the Sensorimotor Functions and Parameter Determination:

Obtaining Laplace Coefficients:

- Methodology: The Laplace coefficients were determined using a combination of system identification techniques and machine learning algorithms, utilizing extensive datasets from both simulated and experimental neural data.
- Process: These coefficients were optimized through a least square fitting procedure, iteratively adjusted to minimize the error between model predictions and observed data, enhancing model accuracy and stability.

Tuning Active/Reactive Parameters:

- Active Parameters: Tuned based on proactive interactions required in active grasping scenarios, using trial-and-error in controlled experimental settings to align closely with human performance metrics.
- Reactive Parameters: Adjusted for quicker responsiveness and higher sensitivity in reactive scenarios, involving dynamic simulations to handle sudden changes effectively.

Determination of Transduction Functions:

- Mathematical Formulation: We selected specific forms of transduction functions, $\frac{a}{S^2+bS+c}$ (active grasping) and $\frac{a}{bS^3+cS^2+dS+e}$ (reactive grasping),

based on their historical success in similar biomechanical models and their ability to comprehensively represent the dynamics of sensory input conversion into motor outputs.

- Empirical Validation: These functions were validated against a subset of data not used in the training phase, ensuring they capture physiological processes accurately without overfitting.
- Data-Driven Approach: Empirical data collected from human subjects and the ATSS during active and reactive grasping tasks provided a rich dataset for system identification, used to extract Laplace coefficients that best represented the relationship between tactile signals and motor neuron activations.
- System Identification and MATLAB Code: The MATLAB code for generating and optimizing transduction functions for both grasping types is included in the supplementary information (Data. S2), enhancing the ability of other researchers to replicate and validate our findings.

Fig. S1.

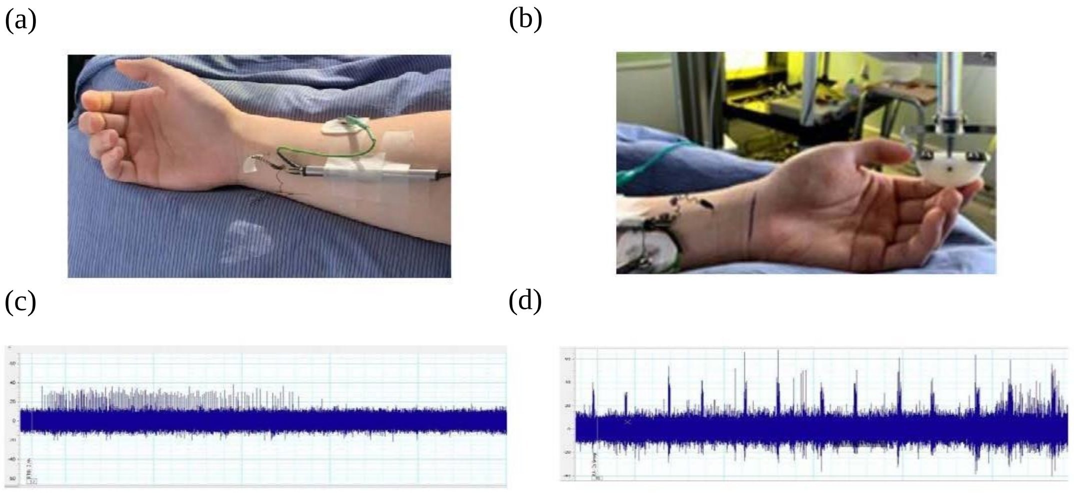

Fig. S1 The microneurography test and the experimental results. (a) Experimental setup for microneurography, the measurement was performed by inserting a tungsten electrode into the median nerve at the wrist. This technique captured single-afferent neural signals, which were essential for assessing the tactile sensitivity and neural response properties of the subjects. The tactile units' receptive fields were systematically stimulated using a Robotic Tactile Stimulator (RTS) to apply controlled sweeping motions across the fields at specified forces. (b) The stimulator is used to activate the cutaneous mechanoreceptor. (c) Example neural dynamics recorded from an SAI tactile unit. (d) Example neural dynamics recorded from a FAI tactile unit

Fig. S2.

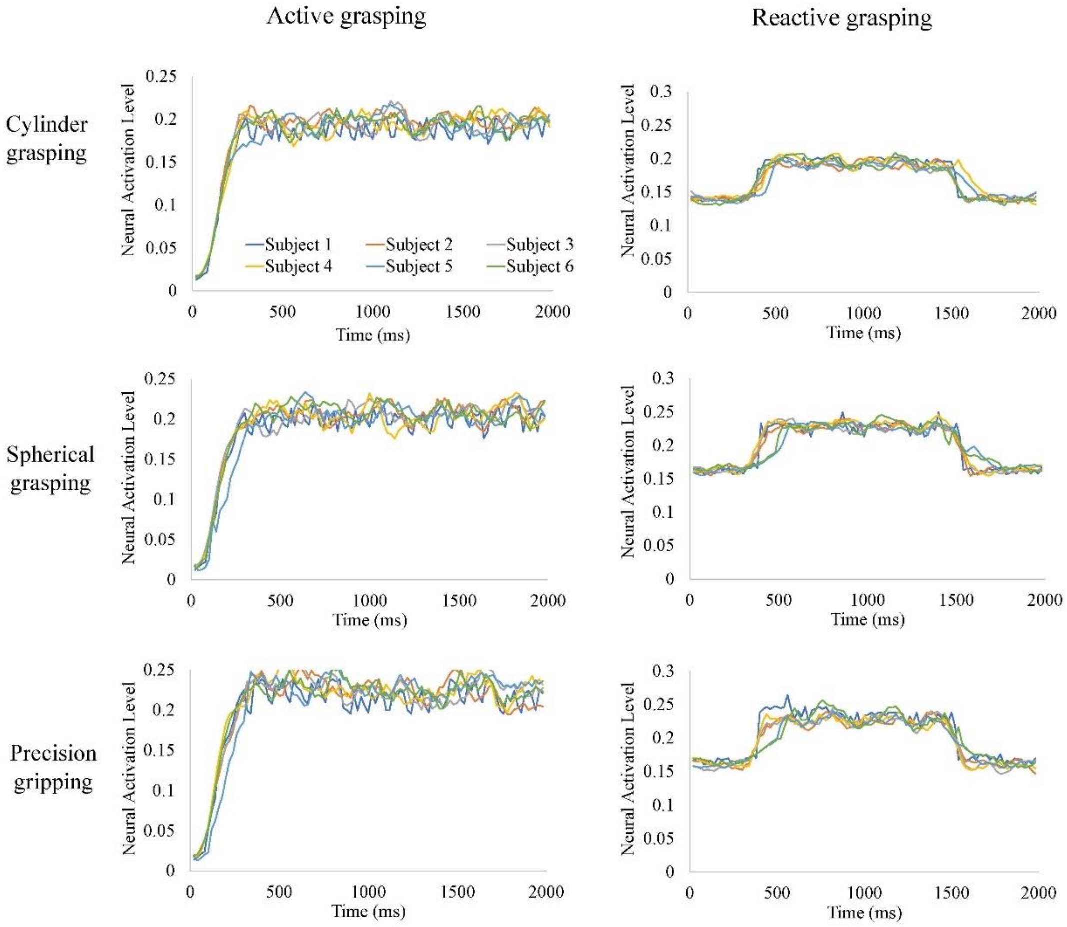

Fig. S2. Experimental results based on all six human subjects. Subject 1 is the 24-year-old male subject employed for developing the multi-level numerical model for computing afferent neural signals and developing the biomimetic hand in this study. The neural activation levels extracted from the EMG signals captured from all six subjects during active and reactive grasping are shown.

Fig. S3.

(a)

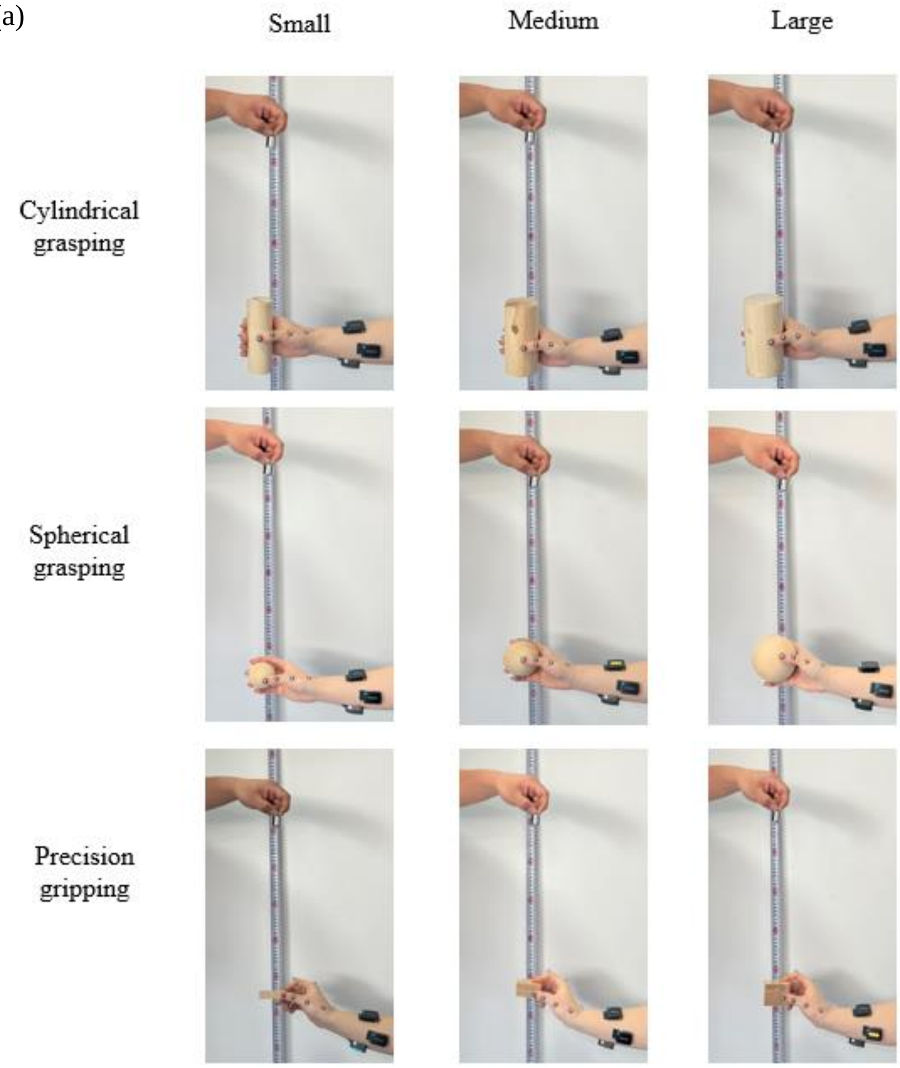
Small
Medium
Large
Cylindrical grasping
Spherical grasping
Precision gripping

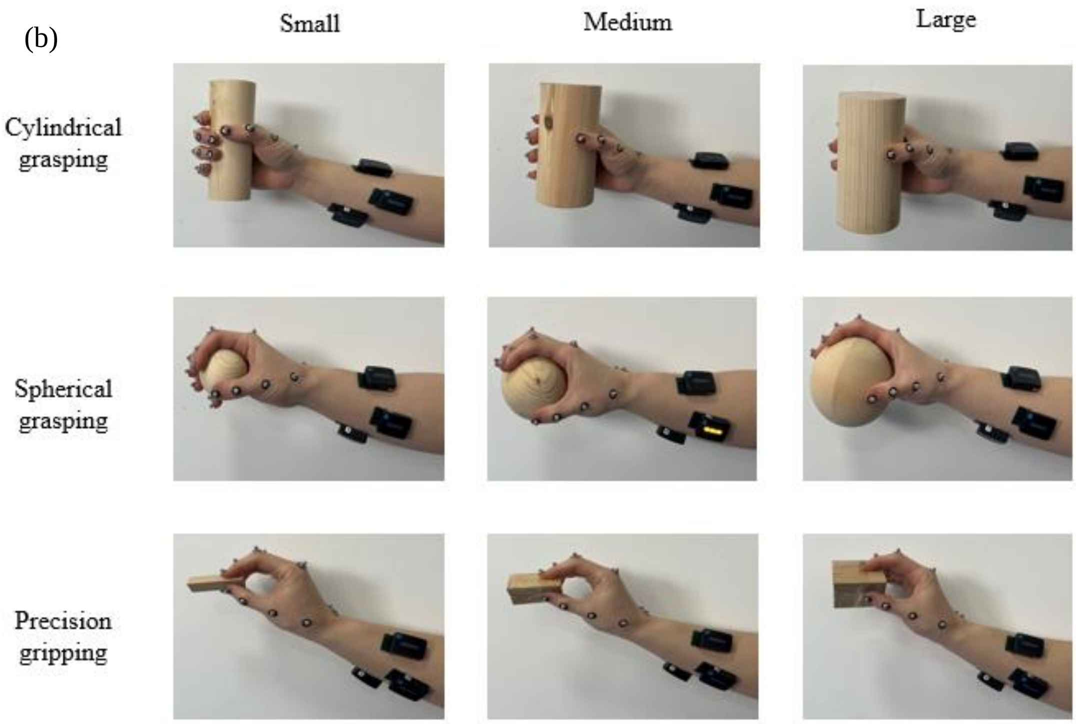

Fig. S3 In-vivo grasping experiment. (a) Active grasping: Human subject grasping and lifting cylinders, spheres, and triangle prisms from a table while markers recorded hand kinematics and electromyography signals. Objects of different sizes (small, medium, and large) were used. (b) Reactive grasping: The subject, blindfolded, lifted a 20g weight to a specified height and released it onto a grasped object, reacting only to tactile stimuli. Various grasping tasks, including cylindrical, spherical, and precision gripping, were performed.

Fig. S4.

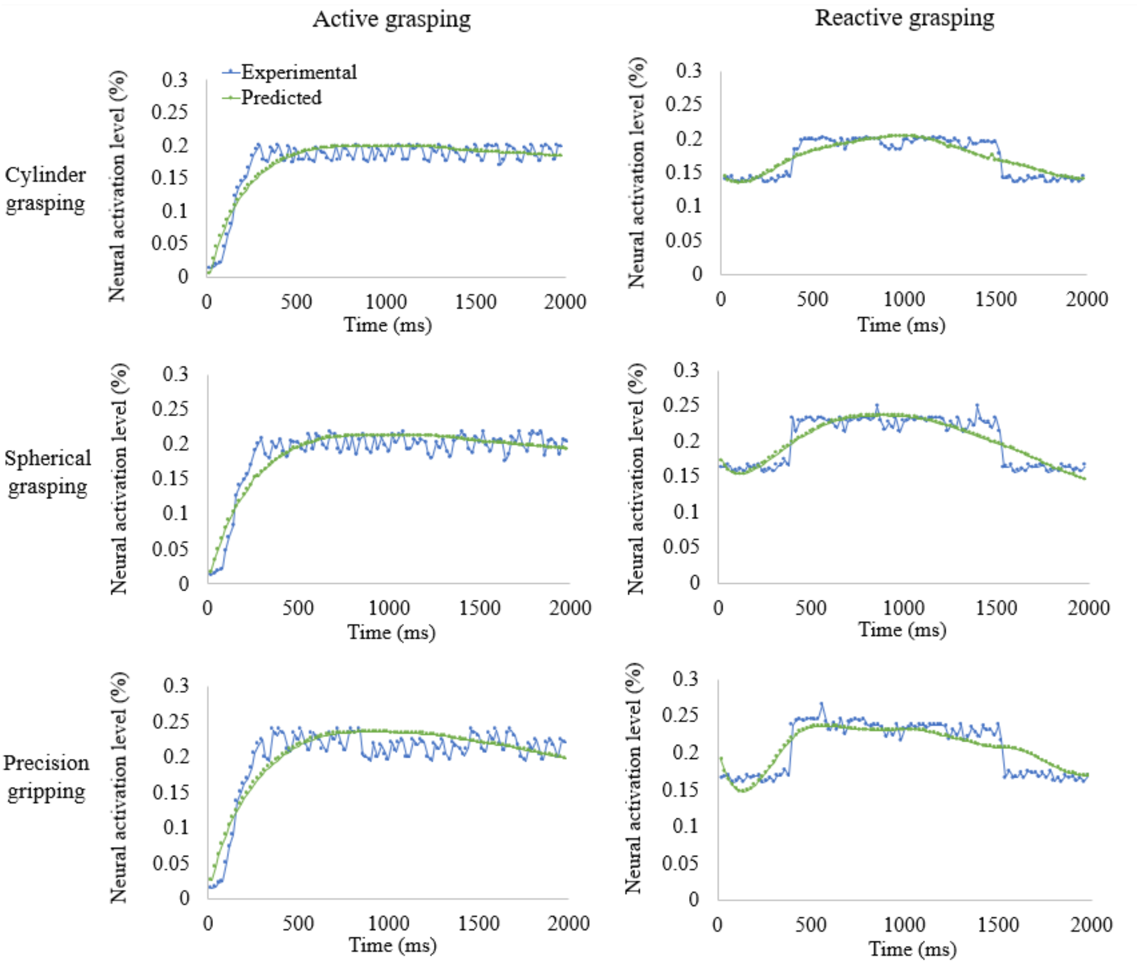

Fig. S4 The neural activation levels predicted based on summarized transduction function compared with those computed based on the electromyography signals captured from the human subject. The activation level over 2s of active and reactive grasping were predicted by input the 2nd order cuneate neural dynamics and summarized transduction function describe the sensory motor control strategy.

Fig. S5.

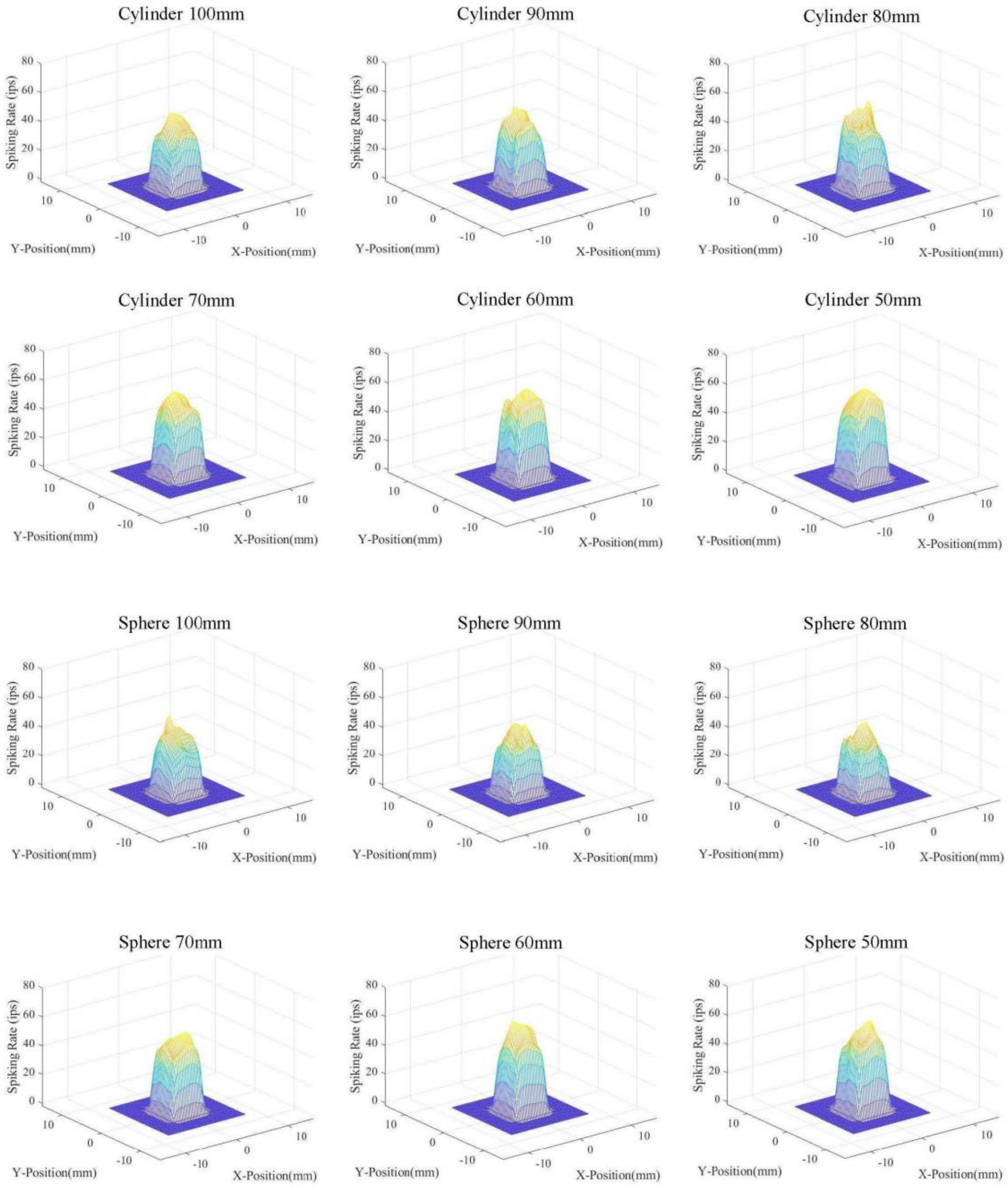

Fig. S5 The firing rates of the neuromorphic tactile signals in terms of SAI tactile units. The distribution of the spiking rate of the neuromorphic tactile signals in terms of SAI tactile units computed over the 6 by 6 tactile sensing elements. The horizontal axis stands for the locations of tactile sensing elements within the contact area, the vertical axis is the spiking rate.

Fig. S6.

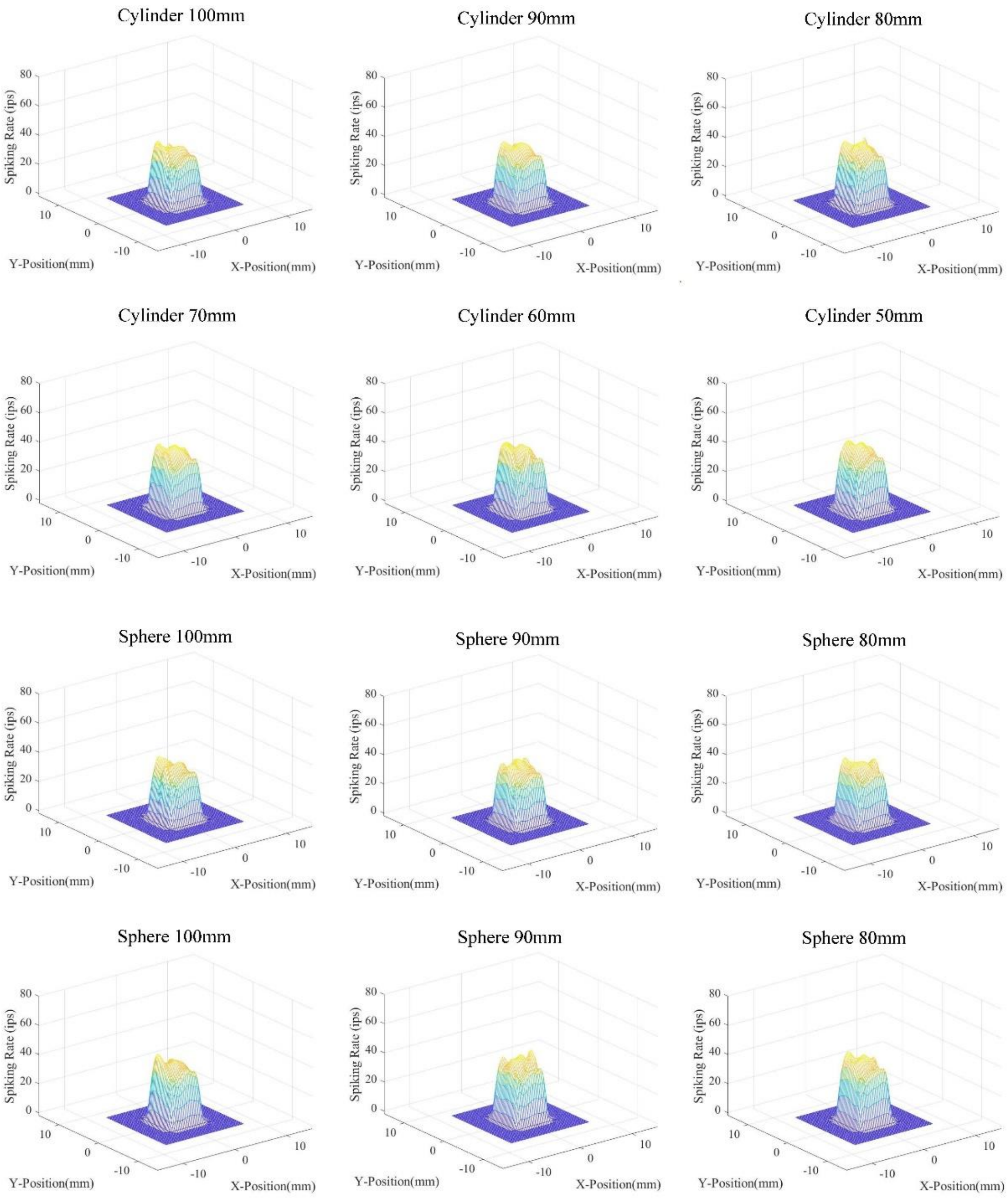

Fig. S6 The firing rates of the neuromorphic tactile signals in terms of FAI tactile units. The distribution of the spiking rate of the neuromorphic tactile signals in terms of FAI tactile units computed over the 6 by 6 tactile sensing elements. The horizontal axis stands for the locations of tactile sensing elements within the contact area, the vertical axis is the spiking rate.

Fig. S7.

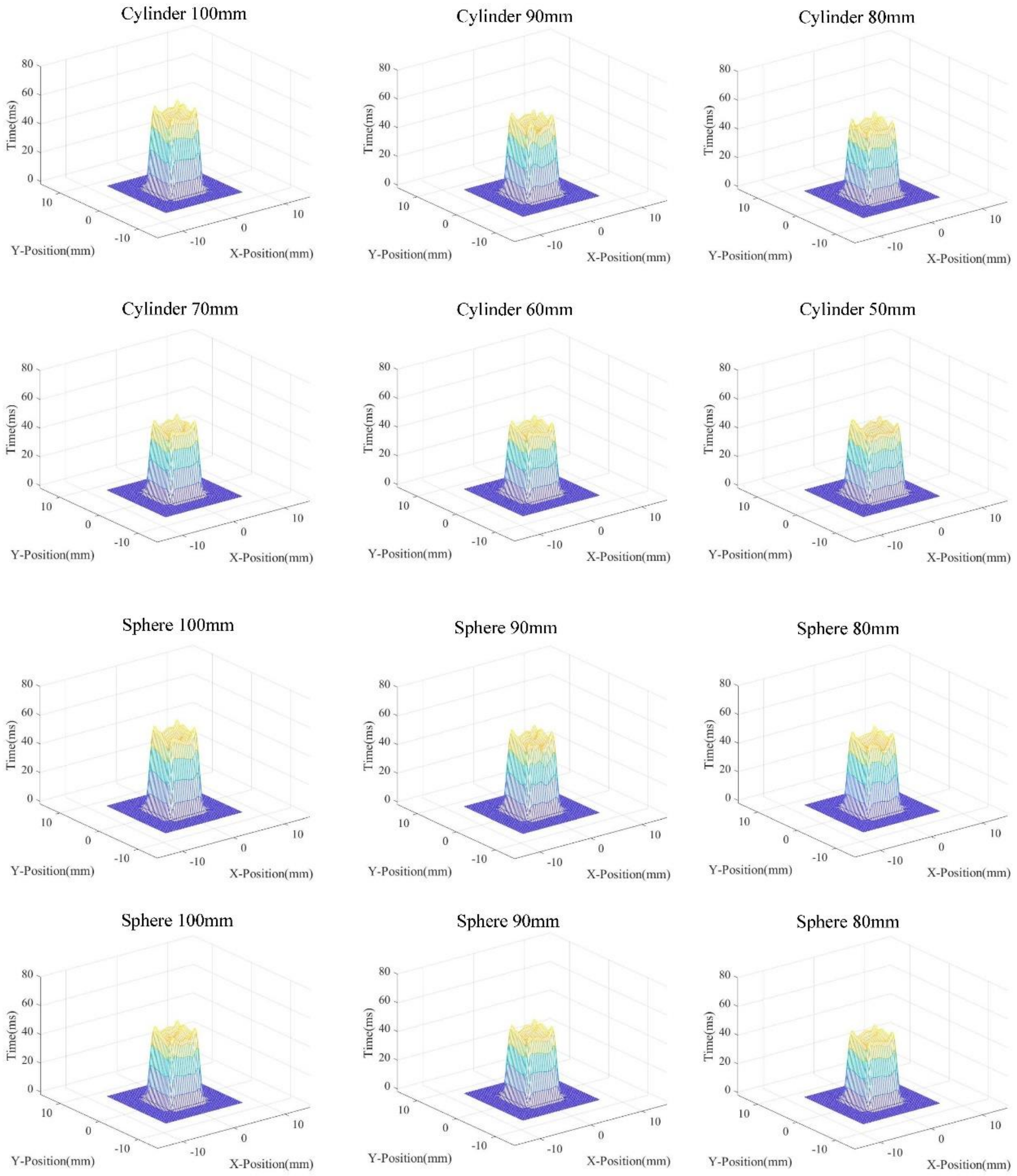

Fig. S7 The first spike latency of the neuromorphic tactile signals in terms of SAI tactile units. The distribution of the first spike latency of the neuromorphic tactile signals in terms of SAI tactile units computed over the 6 by 6 tactile sensing elements. The horizontal axis stands for the locations of tactile sensing elements within the contact area, the vertical axis is the time.

Fig. S8.

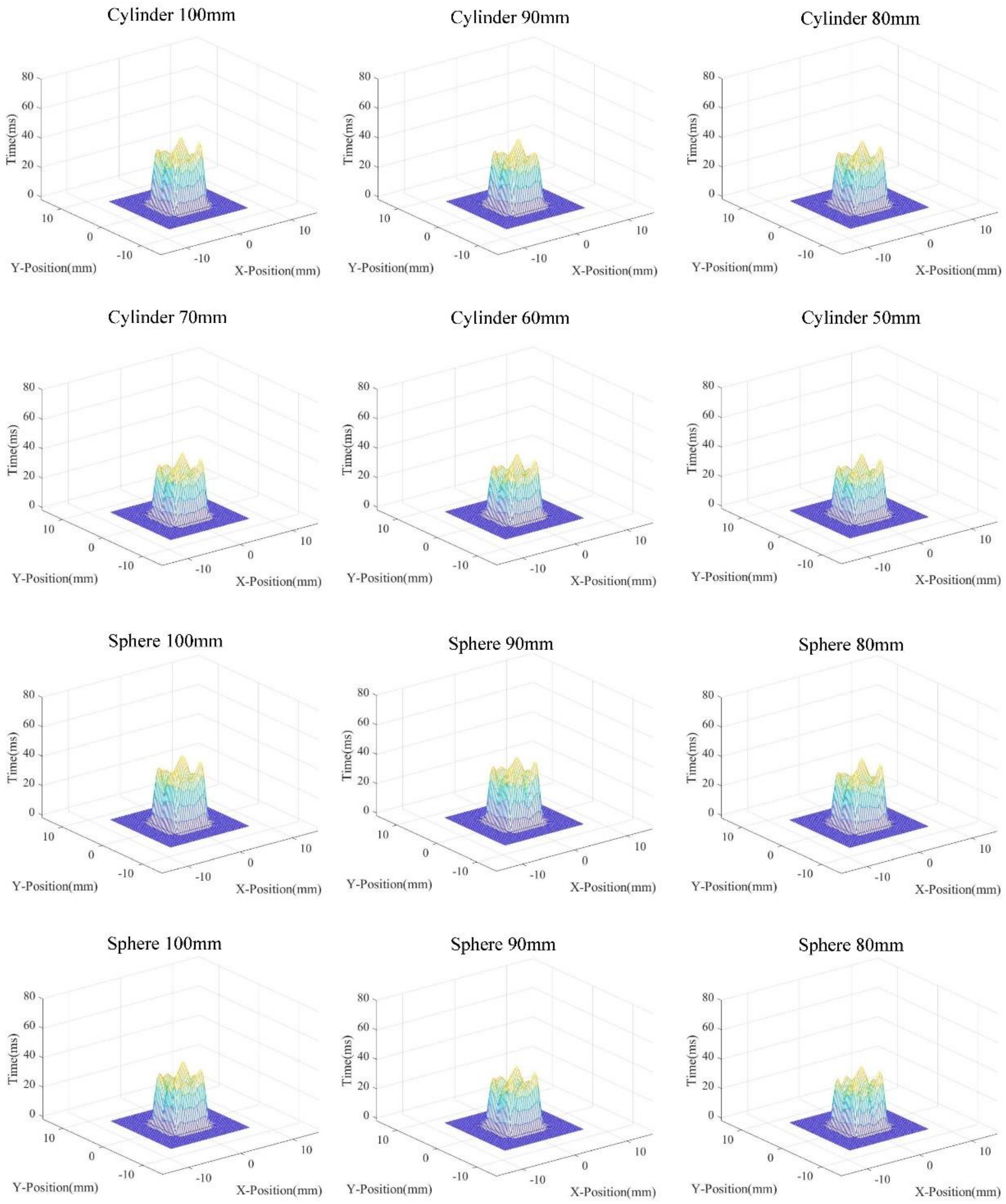

Fig. S8 The first spike latency of the neuromorphic tactile signals in terms of FAI tactile units. The distribution of the first spike latency of the neuromorphic tactile signals in terms of FAI tactile units computed over the 6 by 6 tactile sensing elements. The horizontal axis stands for the locations of tactile sensing elements within the contact area, the vertical axis is the time.

Fig. S9.

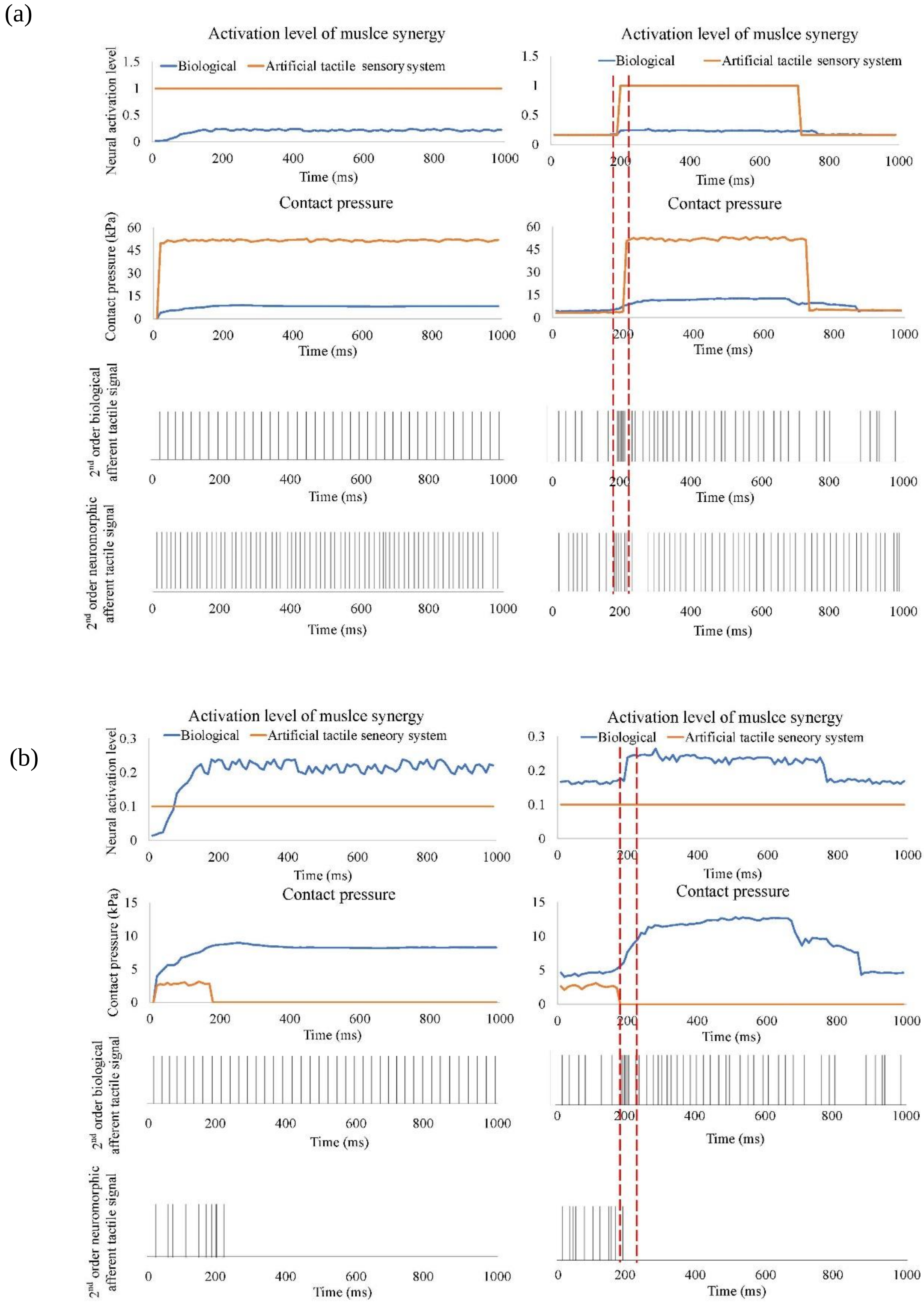

Fig. S9. The neural activation level of muscle synergy and contact pressure during the active and reactive grasping implemented by ATSS. The biological and neuromorphic 2nd order tactile afferent signals are presented here. (a) Contact pressure and afferent tactile signals under the highest neural

activation level or maximum voluntary contraction forces. (b) Contact pressure and afferent tactile signals obtained under 10% of the maximum neural activation level of the muscle synergy. An unstable grasping scenario is depicted where the grasped object detached from the biomimetic hand after an external impact. Consequently, the contact pressure dropped to zero, and no neuromorphic signal was observed following the object's fall.

Fig. S10.

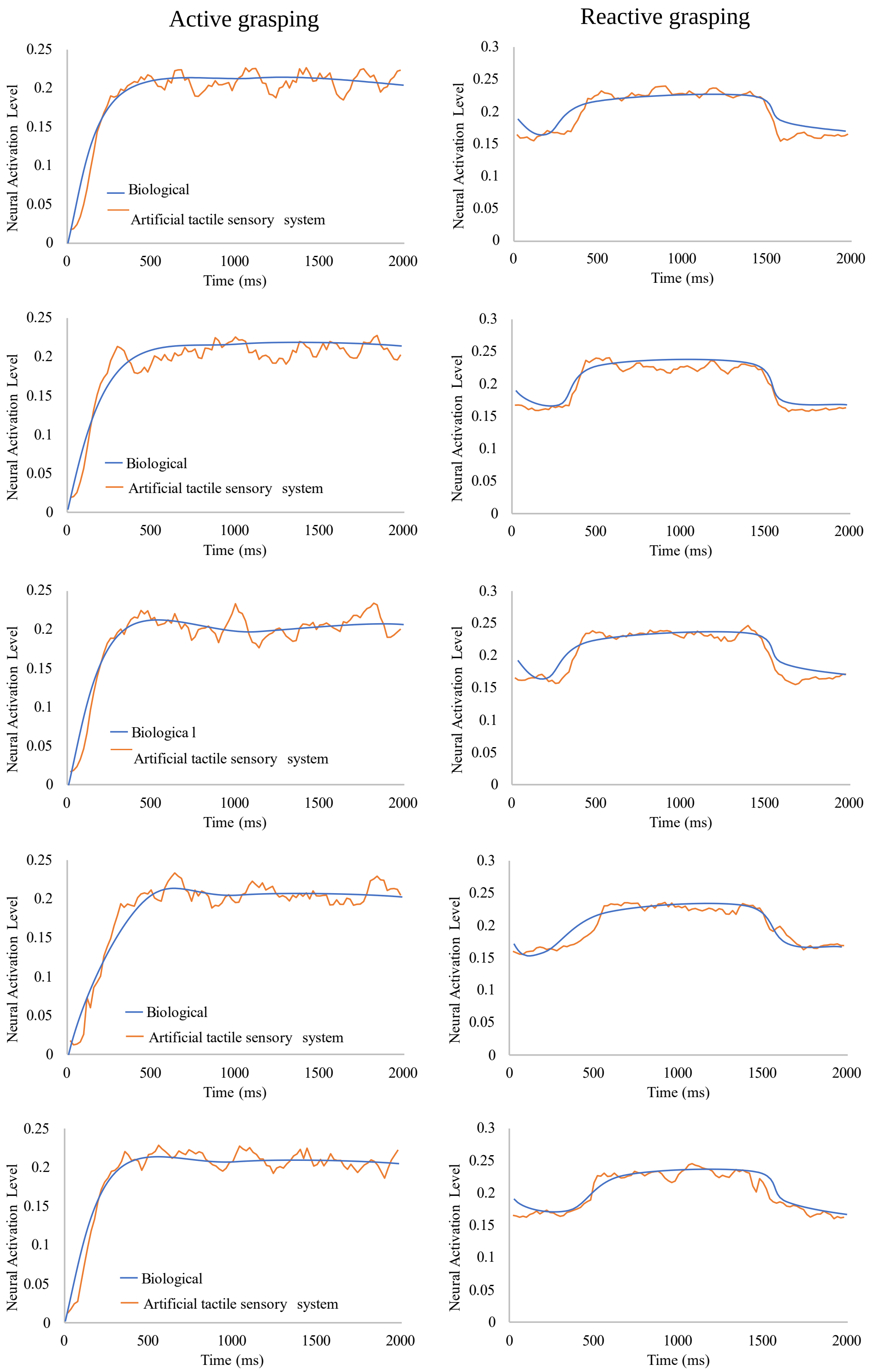

Fig. S10 The measured neural activation levels and those predicted based on summarized transduction function. The data of all the other 5 subjects are presented.

Fig. S11.

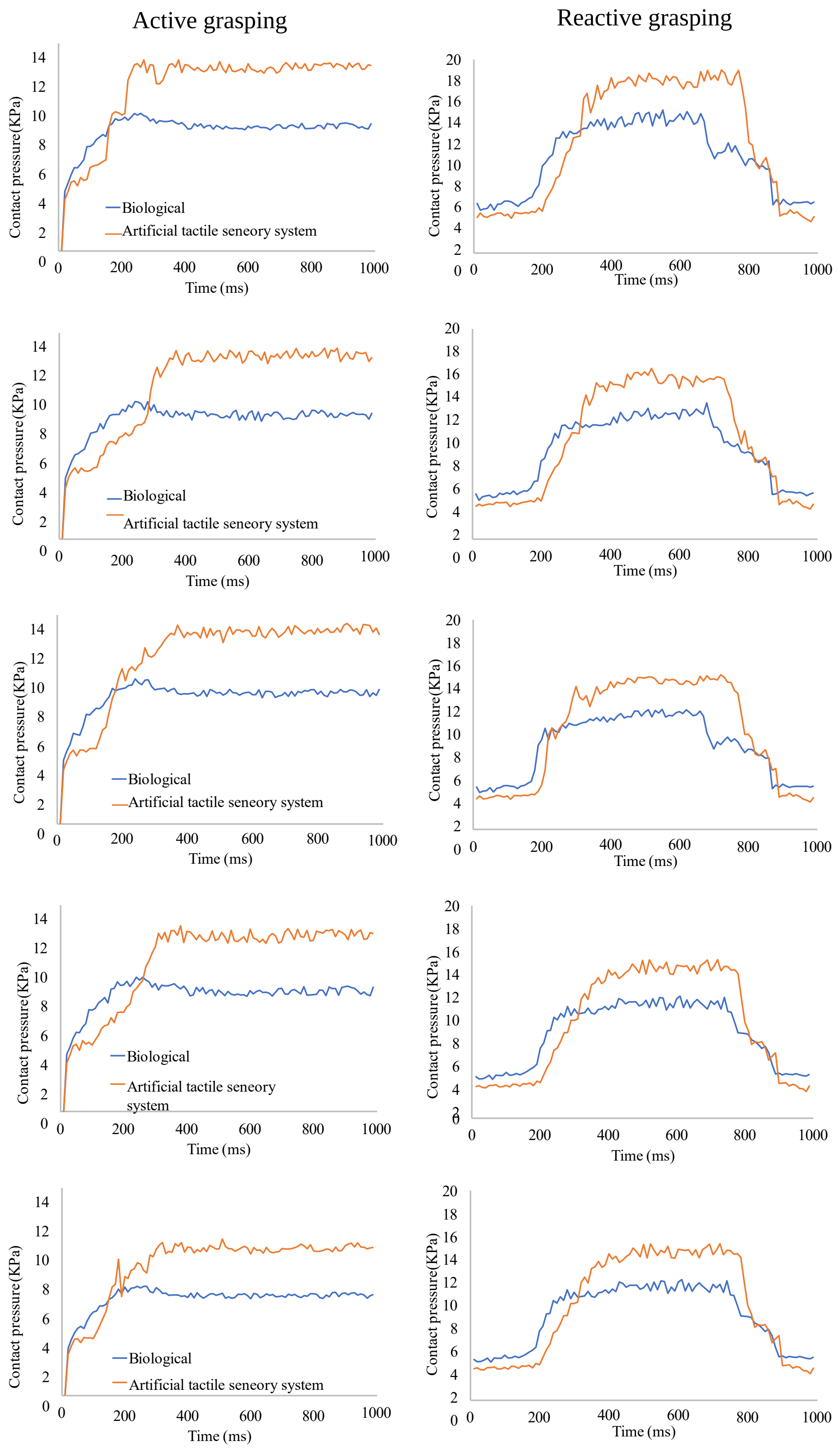

Fig. S11 The contact pressure on the index finger of human and biomimetic hand during active and reactive grasping. The data of all the other 5 subjects are presented.

Fig. S12.

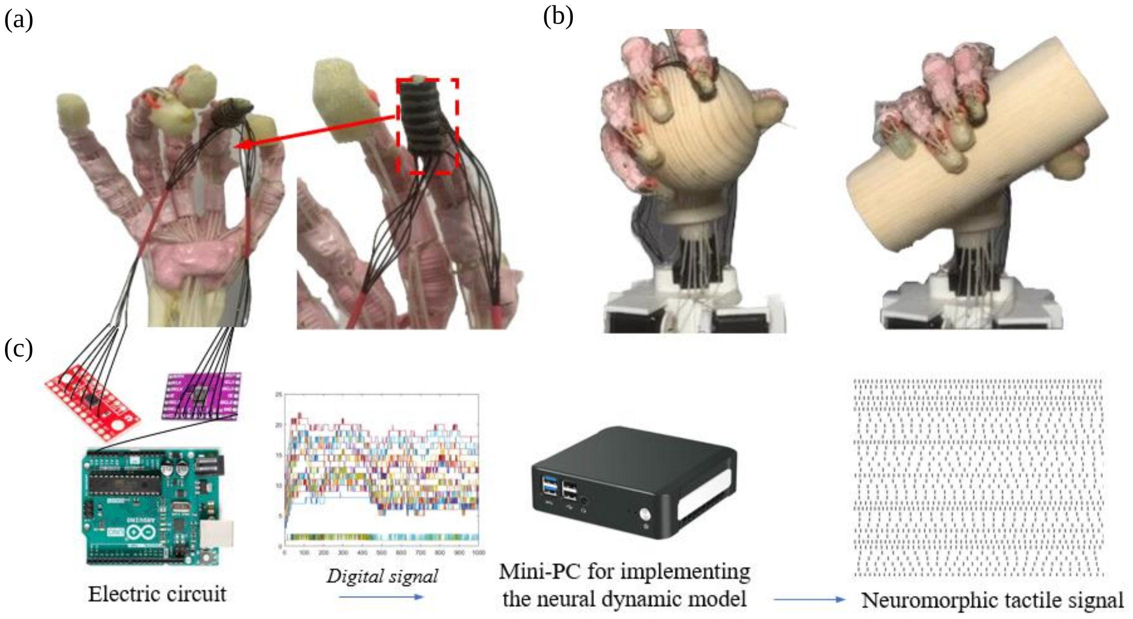

Fig. S12 The hardware setting of the artificial tactile sensory system. (a) The biomimetic hand with a tactile sensor array 3D-printed on the distal index phalanx. (b) Spherical and cylindrical grasping performed by the biomimetic hand without skin. (c) The electrical circuit for processing pressure signals from the tactile sensor. An Arduino Uno board was connected to shift registers and a multiplexer for collecting and converting the analog signals into digital signals. A mini-PC embedded with the neural dynamic model was used to process the digital pressure signals into neuromorphic afferent tactile signals.

Fig. S13.

(a)

(b)

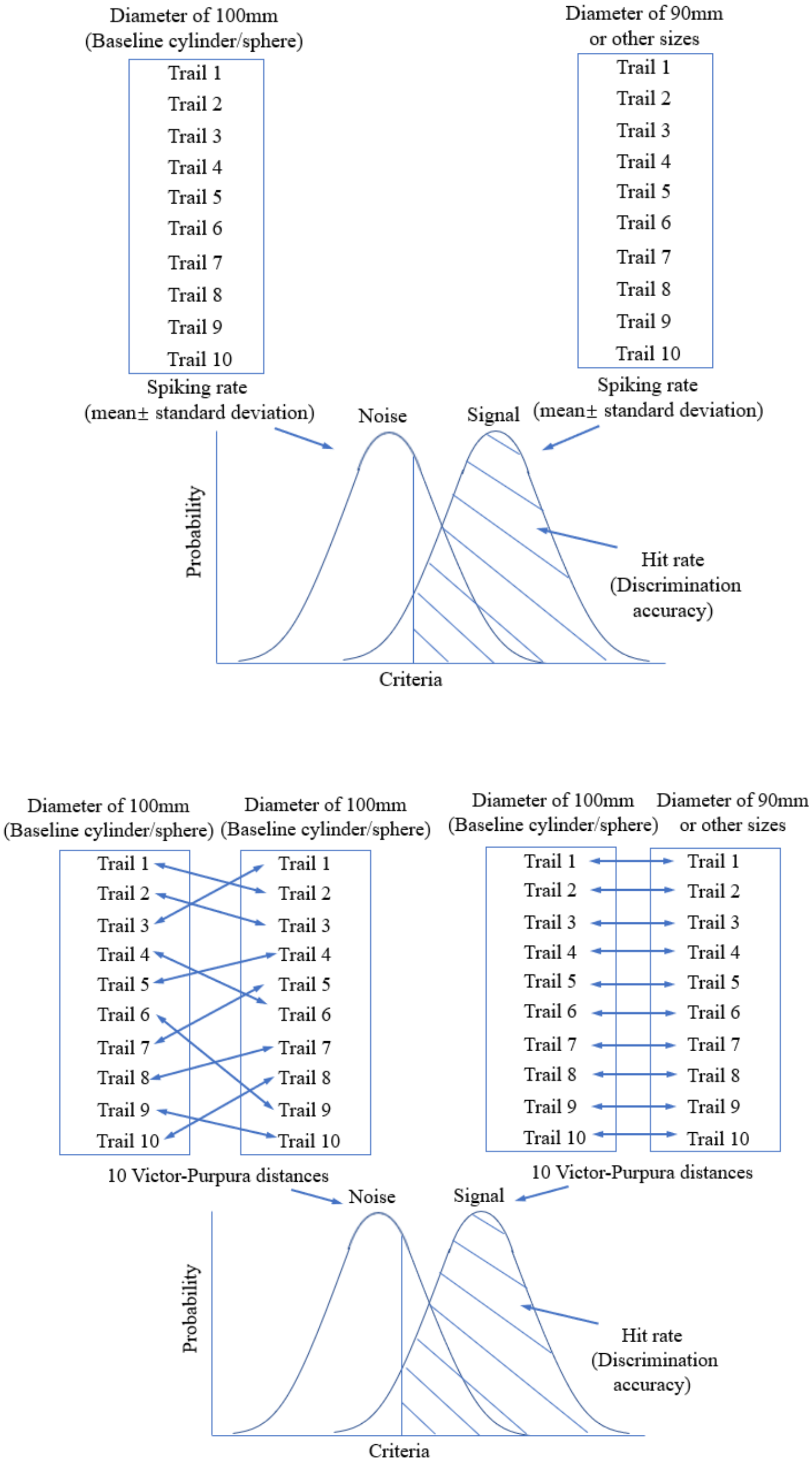

Fig. S13 The computing of discrimination accuracy is based on the neural features of spiking rate and Victor-Purpura distance. (a) Discrimination accuracy computed based on rate coding. The neural dynamics evoked during contact with the baseline object were recorded. The mean and standard

deviation of the spiking rates during 10 touches of the baseline object were summarized as the 'noise' signal in signal detection theory (SDT). Meanwhile, the mean and standard deviation of the spiking rates during contact with other objects, used to differentiate them from the baseline object, were regarded as the 'signal' in SDT. (b) Discrimination accuracy computed based on Victor-Purpura distance. The 'noise' signal in signal detection theory was defined as the Victor-Purpura distance among tactile signals during 10 touches of the baseline object (diameter 100mm). Victor-Purpura distances between neural dynamics evoked when touching the baseline and other objects with diameters less than 100mm were regarded as the 'signal' in SDT to evaluate discrimination accuracy. A total of 100 Victor-Purpura distances were computed. All cylinders were perceptually tested 10 times. The Victor-Purpura distances were calculated for differentiating the neuromorphic tactile signals evoked by contacting cylinders with diameters of 100mm and 90mm, 100mm and 80mm, 100mm and 70mm, 100mm and 60mm, 100mm and 50mm. The same method was used to compute discrimination accuracy for recognizing spheres with different diameters. Therefore, there were a total of 50 Victor-Purpura distances for differentiating cylinders and another 50 for spheres from the baseline objects. For the in-vivo discrimination test, the subject was blindfolded and asked to sit at a table. Cylinders or spheres were presented in pairs, either with the same or different diameters. The subject was required to judge whether the pairs of cylinders or spheres were the same or not. Only the index finger was allowed to touch the objects, and the subject's wrist was fixed. The test was conducted in blocks, with each block containing 20 comparisons (10 pairs of 50mm-50mm objects and 10 pairs of 50mm-60mm or other diameters, spheres or cylinders with all five different diameters presented in each session). The pairs of surfaces varied randomly from block to block. In total, 10 blocks were performed (5 for cylinders and 5 for spheres). The probability of detection was calculated for each cylinder or sphere, and the entire test was repeated 3 times to ensure reliability and generality of the results. Before the test, several practice blocks were conducted to train the subjects and ensure the reliability of the experimental results.

Fig. S14.

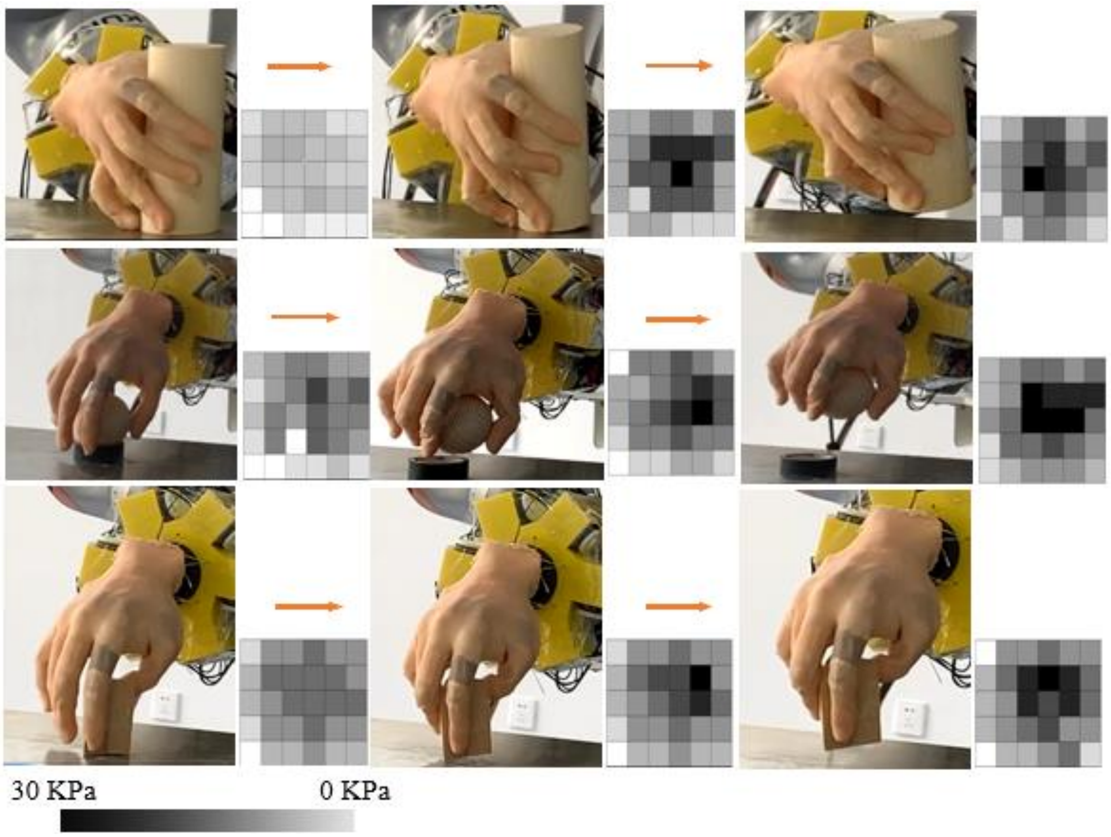

Fig. S14 Cylindrical, spherical grasping and precision gripping were performed by the ATSS. A customized graphical user interface (GUI) was developed to visualize the pressure distribution across the 36 tactile sensing elements mounted on the index of the biomimetic hand. The magnitude of the contact pressure was presented using grayscale. The active grasping was depicted in three stages: before contact, during contact adjustment, and during stable grasping.

Table S1. The values of poles of the transduction function representing active grasping (Trail 1)

Size	Precision gripping	Cylindrical grasping	Spherical grasping
Small	-0.008784216	-0.00682638	-0.005668753
	-0.004143534	-0.003751466	-0.005842522
Medium	-0.007874663	-0.006210732	-0.00436093
	-0.003202536	-0.00351441	-0.004313552
Large	-0.00684563	-0.00506776	-0.002856651
	-0.002559255	-0.002689282	-0.003141594

Table S2. The values of poles of the transduction function representing active grasping (Trail 2)

Size	Precision gripping	Cylindrical grasping	Spherical grasping
Small	-0.008685678	-0.00664576	-0.00586757
	-0.004065756	-0.00369853	-0.00590674
Medium	-0.007767867	-0.006210732	-0.00463467
	-0.003346784	-0.003746867	-0.00474875
Large	-0.00696577	-0.005124354	-0.00290675
	-0.00335746	-0.00274566	-0.00305687

Table S3. The values of poles of the transduction function representing active grasping (Trail 3)

Size	Precision gripping	Cylindrical grasping	Spherical grasping
Small	-0.008566834	-0.006578456	-0.00557345
	-0.003865768	-0.003605346	-0.005735788
Medium	-0.007344556	-0.00579548	-0.00426645
	-0.00297345	-0.00326875	-0.00415645
Large	-0.00646895	-0.00478073	-0.002645783
	-0.00286546	-0.00245732	-0.00285577

Table S4. The values of poles of the transduction function representing active grasping (Trail 4)

Size	Precision gripping	Cylindrical grasping	Spherical grasping
Small	-0.00826898	-0.00726834	-0.005255256
	-0.004345663	-0.00414354	-0.00602465
Medium	-0.007957897	-0.00607568	-0.004046835
	-0.00347696	-0.00394679	-0.004638657
Large	-0.00727783	-0.00547691	-0.002578525
	-0.00306746	-0.00326816	-0.003453416

Table S5. The values of poles of the transduction function representing active grasping (Trail 5)

Size	Precision gripping	Cylindrical grasping	Spherical grasping
Small	-0.008256876	-0.00658678	-0.00536768
	-0.00456856	-0.00396478	-0.00595678
Medium	-0.007874663	-0.006210732	-0.00418794
	-0.003202536	-0.00370479	-0.00445686
Large	-0.00637683	-0.00478378	-0.002689468
	-0.003578937	-0.00295789	-0.00326873

Table S6. The values of poles of the transduction function representing reactive grasping (Trail 1)

Size	Precision gripping	Cylindrical grasping	Spherical grasping
Small	-0.01651144	-0.027385004	-0.016888312
	-0.001634424	-0.000927904	-0.00375288
	0.011566456	0.006123652	0.013985422
Medium	-0.01531046	-0.024251904	-0.014439256
	-0.001377552	-0.000625522	-0.00254328
	0.010083395	0.005910711	0.01316537
Large	-0.01373746	-0.021380905	-0.012309367
	-0.001145848	-0.000426679	-0.00132144
	0.01563545	0.000736416	0.014695521

Table S7. The values of poles of the transduction function representing reactive grasping (Trail 2)

Size	Precision gripping	Cylindrical grasping	Spherical grasping
Small	-0.018352421	-0.031056253	-0.01763252
	-0.001761632	-0.001138205	-0.00416052
	0.012515625	0.00706522	0.015069241
Medium	-0.017569525	-0.027296232	-0.01635241
	-0.001426582	-0.001194522	-0.0028602
	0.011653006	0.006291475	0.014925411
Large	-0.015635226	-0.02390054	-0.013526241
	-0.001262466	-0.000562342	-0.001563521
	0.017523696	0.000923852	0.016752632

Table S8. The values of poles of the transduction function representing reactive grasping (Trail 3)

Size	Precision gripping	Cylindrical grasping	Spherical grasping
Small	-0.01762525	-0.029526041	-0.016888312
	-0.001752324	-0.000927904	-0.00375288
	0.01206221	0.006123652	0.014262005
Medium	-0.016721605	-0.024251904	-0.015232052
	-0.001410625	-0.000625522	-0.002705621
	0.012620982	0.005910711	0.015252452
Large	-0.014160663	-0.021380905	-0.013056545
	-0.001350457	-0.000426679	-0.001546586
	0.016106235	0.000736416	0.012182547

Table S9. The values of poles of the transduction function representing reactive grasping (Trail 4)

Size	Precision gripping	Cylindrical grasping	Spherical grasping
Small	-0.014412963	-0.024012336	-0.015984216
	-0.001466923	-0.000851053	-0.003501365
	0.010362521	0.005801547	0.012810542
Medium	-0.014262582	-0.02120136	-0.013980147
	-0.001377552	-0.000590333	-0.002102681
	0.009153621	0.005510214	0.012001562
Large	-0.012893025	-0.019845633	-0.011930217
	-0.001004153	-0.000400133	-0.00132144
	0.014580521	0.000685405	0.01381652

Table S10. The values of poles of the transduction function representing reactive grasping (Trail 5)

Size	Precision gripping	Cylindrical grasping	Spherical grasping
Small	-0.017013652	-0.029253625	-0.017963521
	-0.001762141	-0.001003266	-0.004163205
	0.013015625	0.00656352	0.01426352
Medium	-0.016321101	-0.0285232	-0.016223521
	-0.001413601	-0.000712232	-0.00352423
	0.01266202	0.006982336	0.01316537
Large	-0.014011034	-0.025897145	-0.014652025
	-0.001293552	-0.000426679	-0.001932052
	0.016954241	0.000736416	0.015793611

Table S11. The values of poles of the transduction function representing active grasping (Subject 2)

Size	Precision gripping	Cylindrical grasping	Spherical grasping
Small	-0.00876478	-0.00747956	-0.00583897
	-0.00442224	-0.00349164	-0.00559737
Medium	-0.007602	-0.00644785	-0.00418105
	-0.00320054	-0.00384372	-0.0040414
Large	-0.00760713	-0.00471107	-0.00296367
	-0.00289019	-0.00275717	-0.00351499

Table S12. The values of poles of the transduction function representing reactive grasping (Subject 2)

Size	Precision gripping	Cylindrical grasping	Spherical grasping
Small	-0.01434379	-0.02445892	-0.01720732
	-0.00153657	-0.00081638	-0.00363759
	0.00960861	0.00583214	0.01426828
Medium	-0.0157074	-0.02366804	-0.01340161
	-0.00125152	-0.00056845	-0.00224706
	0.00889276	0.00561156	0.01244943
Large	-0.0117558	-0.02214549	-0.01125253
	-0.00110108	-0.00040639	-0.00141712
	0.01548845	0.0007317	0.01296237

Table S13. The values of poles of the transduction function representing active grasping (Subject 3)

Size	Precision gripping	Cylindrical grasping	Spherical grasping
Small	-0.00808178	-0.00637429	-0.00510639
	-0.00431462	-0.00361377	-0.00560649
Medium	-0.00833757	-0.0056263	-0.00411824
	-0.0031521	-0.00381392	-0.00470524
Large	-0.00744633	-0.00468436	-0.00306132
	-0.00252631	-0.00295207	-0.00324209

Table S14. The values of poles of the transduction function representing reactive grasping (Subject 3)

Size	Precision gripping	Cylindrical grasping	Spherical grasping
Small	-0.01384522	-0.02310975	-0.01646384
	-0.00160694	-0.00093303	-0.003937
	0.01043191	0.00563498	0.01228078
Medium	-0.01302713	-0.02129348	-0.0126248
	-0.00147919	-0.00062105	-0.00193553
	0.00864855	0.00510217	0.01117102
Large	-0.01268831	-0.02012814	-0.01200206
	-0.00101069	-0.00041707	-0.00149027
	0.0160281	0.00064167	0.0149236

Table S15. The values of poles of the transduction function representing active grasping (Subject 4)

Size	Precision gripping	Cylindrical grasping	Spherical grasping
Small	-0.00920599	-0.00762776	-0.00611233
	-0.00458138	-0.00338849	-0.0056438
Medium	-0.00712007	-0.00668081	-0.00478154
	-0.00343583	-0.0033138	-0.00390551
Large	-0.00739324	-0.00565713	-0.00287273
	-0.00277564	-0.00262879	-0.00342936

Table S16. The values of poles of the transduction function representing reactive grasping (Subject 4)

Size	Precision gripping	Cylindrical grasping	Spherical grasping
Small	-0.0151246	-0.02168844	-0.01610806
	-0.00143639	-0.00087319	-0.0036039
	0.0110689	0.00593762	0.01167909
Medium	-0.01591656	-0.02360289	-0.01564099
	-0.00141548	-0.00055371	-0.0019295
	0.00930048	0.00543201	0.01157926
Large	-0.01188538	-0.01859589	-0.01344989
	-0.00109846	-0.00045184	-0.00133093
	0.01562968	0.00070253	0.01305374

Table S17. The values of poles of the transduction function representing active grasping (Subject 5)

Size	Precision gripping	Cylindrical grasping	Spherical grasping
Small	-0.00855675	-0.00615947	-0.00562173
	-0.00442025	-0.00397013	-0.00568234
Medium	-0.0088599	-0.00622029	-0.00433681
	-0.00300323	-0.00360943	-0.00442912
Large	-0.00728923	-0.00499587	-0.00266197
	-0.00231285	-0.00286162	-0.00313788

Table S18. The values of poles of the transduction function representing reactive grasping (Subject 5)

Size	Precision gripping	Cylindrical grasping	Spherical grasping
Small	-0.01576694	-0.02528217	-0.01741824
	-0.00164738	-0.00078604	-0.00337442
	0.01147464	0.00640508	0.01184803
Medium	-0.01454694	-0.02074038	-0.01323565
	-0.00154226	-0.00065554	-0.00225183
	0.00864246	0.00556444	0.01259627
Large	-0.01301408	-0.01924963	-0.01220847
	-0.00106695	-0.00037047	-0.00139198
	0.0160975	0.00075262	0.01394304

Table S19. The values of poles of the transduction function representing active grasping (Subject 6)

Size	Precision gripping	Cylindrical grasping	Spherical grasping
Small	-0.00829641	-0.00681749	-0.00565931
	-0.00449248	-0.00385892	-0.00541746
Medium	-0.00848097	-0.00686789	-0.00479678
	-0.00302465	-0.00359082	-0.00430936
Large	-0.00626659	-0.00528094	-0.00264824
	-0.00233056	-0.0026246	-0.00302531

Table S20. The values of poles of the transduction function representing reactive grasping (Subject 6)

Size	Precision gripping	Cylindrical grasping	Spherical grasping
Small	-0.01409258	-0.02481368	-0.01761862
	-0.00138152	-0.00094552	-0.00385713
	0.00971933	0.00647545	0.01243121
Medium	-0.01443818	-0.01908828	-0.01345269
	-0.00152185	-0.00063869	-0.00229038
	0.01022393	0.00542171	0.01144348
Large	-0.01182244	-0.0184308	-0.01089382
	-0.00098073	-0.00039482	-0.00143523
	0.01480663	0.00065323	0.01398653

Table S21. The meta data for the 6 subjects involved in the experiment

Number	Gender	Age
1	Male	23
2	Female	28
3	Male	35
4	Female	26
5	Male	43
6	Male	29

Table S22. Victor-Purpura Distances Between Baseline Cylinder/Spherical Object (Diameter: 100mm) and Others. Each object underwent 10 touches, and Victor-Purpura distances were computed between cylinders/spheres with varying diameters (ranging from 50mm to 90mm) and the baseline diameter of 100mm. Additionally, Victor-Purpura distances among the 10 trials of touching the baseline cylinders/sphere were calculated and utilized as 'noise' for signal detection theory to calculate the hit rate. This table presents the average Victor-Purpura distances along with their standard deviations.

Size (mm)	Cylinder	Sphere
100-100	7 (2.6)	9 (3.1)
90-100	19 (4.7)	20 (6.2)
80-100	21 (5.4)	27 (3.5)
70-100	29 (4.4)	34 (7.2)
60-100	38 (6.3)	42 (7.4)
50-100	40 (7.1)	48 (7.6)

References

1 Wei, Y., Zou, Z., Wei, G., Ren, L. & Qian, Z. Subject-specific finite element modelling of the human hand complex: muscle-driven simulations and experimental validation. Annals of Biomedical Engineering **48**, 1181-1195 (2020).
