# Supplementary material for: Human tactile sensing and sensorimotor mechanism: from afferent tactile signals to efferent motor control
Source: Nat Commun. 2024 Aug 10;15:6857. doi: 10.1038/s41467-024-50616-2 (PMC11316806; doi:10.1038/s41467-024-50616-2)
Supplement: Supplementary file 3 — Description Of Additional Supplementary File [file 41467_2024_50616_MOESM3_ESM.pdf]

Description of Additional supplementary file

Movie S1. The sensorimotor performances of the biomimetic hand, modulated by the sensorimotor

Movie S2. Active grasping performed by the artificial tactile sensory system under sensorimotor control algorithm.

Data. S1. The MATLAB code for computing the Victor-Purpura distance.

Data. S2. The MATLAB code for deriving the sensorimotor transduction functions.

Data. S3. The python code for restoring human-like sensorimotor performance on the artificial tactile sensory system.
